# Some Points Obtained by a Three Months Sojourn in New York City

**Published:** 1893-02

**Authors:** W. C. Day

**Affiliations:** Winchester, Ill.


### SOME POINTS OBTAINED BY A THREE MONTHS SOJOURN IN NEW YORK CITY.

BY W. C. DAY, M. D.,
WINCHESTER, ILL.

At the Polyclinic and the other numerous hospitals I witnessed nearly all the important surgical operations, such as amputations, ligation of arteries, resections of joints, tenotomies, gastrotomies, gastrostomies, perineorrhaphies, trachelorrhaphies, operations upon the rectum, upon bones of extremities, upon the cranium, in which the brain and its membranes were fearlessly invaded, and with results almost invariably good; extirpations of mamma, and in fact many and varied surgical procedures requiring too much time to mention.

Beside all the operations at the hospitals I witnessed upon the living, I received at the Polyclinic the most valuable instruction from Professor R. H. M. Dawbarn in operative surgery upon the cadaver.

His class operates under his critical observation and direction, which gives them familiarity in all the principal surgical operations, as well as the proper methods of handling instruments. In this course the technique of all surgical procedures, with the knowledge pertaining to strict antiseptic precautions, are elaborately taught by this brilliant operator and instructor.

I had the pleasure of devoting a great deal of attention to the diseases of the eye, ear, nose and throat. The clinical advantages in those fields are exceedingly valuable and rich to those desirous of adding to their stock of knowledge in such specialties. At the Manhattan Eye and Ear Infirmary there are from one hundred to one hundred and fifty cases of those diseases treated and operated upon daily by the best clinical teachers in the United States.

The subject of anæsthesia is of great practical interest to us all. Ether was used more than nine out of ten times. It was usually administered from the Ormsby inhaler.

The apparatus consists of a rubber mouth and nose cover, a wire wicker sponge-holder, and over this a rubber balloon, which latter is a device originating with the English enhaler known as Clover's.

It is intended by this special apparatus to furnish to the inspired air ether that has been warmed by the expired air.

The vapor being thus warmed, a great many dangers of bringing this volatile cold fluid in contact with the respiratory tract are lessened and possibly avoided. Expired air has a temperature of 95 degrees, and when it is mixed with the ether in the rubber balloon of the inhaler it warms the anæsthetic. The expired air is breathed over and over again with the warmed ether-vapor, so that there is added to the ether narcosis an element of carbon dioxide asphyxia. With quite an extensive experience with the Ormsby inhaler, Professor Wyeth says there is comparatively no danger from its use, and the time required to induce narcosis is very much abridged, and a very much smaller quantity is required. Chloroform is used mostly with children and adults where there is disease of the respiratory organs. It is always administered with an Esmarch's inhaler, taking care to have a due admixture of atmospheric air. Anæsthetics are never given, except in emergencies, without first clearing out the alimentary tract, and prohibiting food for about six hours on the day of the operation; also, very frequently, a hypodermic injection of morphine before the anæsthetic.

Aseptic and antiseptic measures are carried out very thoroughly and elaborately. The patient is given two or three baths previous to an operation, and the surface of the parts to be operated upon is thoroughly shaven, as well as contiguous parts. At least twenty-four hours before operating the site of the operation is covered over by many layers of bichloride wet gauze, which are kept in situ until the patient is anæsthetized. The gauze is then removed, and another thorough washing with brush and soap is given the parts, then the field of operation is sometimes bathed in a solution of iodoform and ether, or permanganate of potassium, followed by oxalic acid. The surgeon, with all his assistants, scrub their hands and arms with brush and soap in running water for many minutes, care always being taken to cleanse thoroughly underneath the finger nails. The hands are then bathed in a solution of bichloride, or else saturated permanganate with subsequent oxalic acid solution, also saturated for a few moments and then rinsed with boiled water. Surgeon and assistants wear clean white gowns, and over the patient there are spread a great many towels wrung out of 1-2000 bichloride solution. If a part is to be irrigated, Thiersch's solution is often used, the formula of which is 4 grs. boric acid and 1 gr. salicylic acid to the ounce of sterilized water.

I was highly impressed with the Hilton-Roser method of opening abscesses, situated in localities where great caution is to be observed on account of nerves and blood vessels. We can best illustrate the technique of the operation by the side of the neck as an example. Grant there is a very large and painful swelling with heat and redness in this locality. The presumption would be that there is an abscess down deep in a net-work of blood vessels and nerves that would make the most daring and reckless surgeon hesitate to plunge a knife into it.

In such a case the abscess can be opened without any danger of wounding a vessel or nerve by proceeding in accordance with the rules given by Hilton and Roser. The first step is to palpate in order to detect fluctuation, which cannot always be determined. After this procedure take a small probe and tap the parts with its blunt end with gentle force all over the swollen and tender region. When the tenderest spot has been touched it can safely be located as the center of the abscess. The next step is to make a small incision in the skin and only through it, not deeper, and through this opening introduce a small aspirator needle cautiously and slowly until the *tactus eruditus* gives evidence of a cavity. Then aspirate for the fluid. If pus flows into the glass barrel of the syringe, the next step is to unscrew the needle from the barrel, and by the side of the needle introduce a grooved director until the point has reached the pus cavity. Next remove the needle and push a pair of scissor-handled dressing forceps down the grove of the director, and when the cavity has been reached remove director. Now dilate the blades of the forceps considerably and forcibly and draw them out of the wound with the blades still widely separated.

The cavity will be emptied by this procedure, and is next irrigated through a drainage tube carried in by the forceps reintroduced for this purpose, which tube is then left in place for a few days.

The real and exact pathological condition in Colles's fracture has not been thoroughly comprehended until quite recently. Dr. Pilcher, of Brooklyn, was the first to give a true description of the position of the bone fragments in that peculiar and perplexing fracture, and to suggest the plan of treatment which if carefully carried out will insure immunity against deformity.

In this fracture the lower fragment is tilted upward and locked against the upper fragment. The fragments are very firmly fixed in this position and are with great difficulty replaced.

The patient should invariably be anæsthetized, otherwise, he unintentionally opposes reduction by rigid tendons and muscles.

Then reduce the fracture by bending the lower fragment backward until the fragments are completely unlocked, afterward bring them into line by forcible traction and kneeding. After the fracture is certainly reduced and no evidence of silver fork deformity exists, almost any kind of retentive apparatus firmly applied will hold the fragments in their normal position, and will produce a good result. Hence the pistol shaped splint is now an exploded idea. The preceding was the invariable practice pursued at the metropolis, and out of a great many such cases which came under my observation I can testify to most excellent results.

A new method of reducing a subglenoid dislocation of the shoulder joint is *Kocher's.*

By this method the shoulder can usually be reduced without anæsthesia and without great pain to the patient; however, here, too, anæsthesia makes the result much more certain.

Bend the arm to a right angle and place the elbow against the side, then turn the hand and arm outward as far as they will go, and whilst in this position bring the arm forward until it reaches the median line. This will open wide the rent in the capsule. Now force the hand back on the *opposite* shoulder, in which manner it will almost invariably slip in place and generally this result occurs before the final motion is made.

Internal hemorrhoids were generally operated upon by clamp and Paquelin's thermocautery. In all the hospitals under my observation this method was practiced. The patient was carefully prepared, by clearing out the alimentary canal, thoroughly cleansing the anal region. The sphincter ani was thoroughly dilated. This step is imperative for the relief of pain, *post operationem.* The rectum was scrupulously cleansed with sponge and soap.

After the operation a rubber tube covered with iodoform gauze was introduced into the rectum about three inches, to enable patient to pass flatus, and it was retained in position by an abundance of iodoform gauze and a T bandage.

The terms typhlitis, perityphlitis and paratyphlitis have been dropped from the nomenclature of diseases by the Academy of Medicine of New York, and the more correct term, appendicitis, substituted.

Appendicitis is a disease belonging exclusively to the domain of surgery, and since it has received the attention commensurate with its gravity many lives have been saved that otherwise were lost. I saw a great many cases operated upon, and every instance proved the wisdom of surgical interference. The relief was prompt and gratifying.

The method of aeration of tissues marks a triumphal advance in surgery, where cautious and accurate dissections are required. It consists in raising the delicate tissues with a pair of dissecting forceps, aided by an assistant with a second pair of forceps, which allows the air to pass into the cellular spaces, rendering the parts elevated nearly transparent. By this procedure the surgeon can cut the transparent tissues without fear of wounding a collapsed and thin vein, which can be distinguished from the transparent fascia. In operations upon the abdominal cavity, in hernia and in the neck regions, where cautious dissections should be made, it has largely supplanted dependence on the grooved director.

Thiersch's skin grafting has proved to be a great boon in the rapid healing of large ulcers and burns, or where a large amount of integument has been removed by surgical procedure or otherwise, in which the edges cannot be brought into apposition.

The method consists in removing the granulations from surface to be grafted by scalpel, levelling it and staunching the blood (but not by ligation) so as to prevent oozing. The cuticle is taken from the thigh generally or arm or back. The parts are well disinfected twenty-four hours previously, and a very thin slice of cuticle, as wide and long as possible, is pared off with a razor. Each slice is placed cautiously and evenly over the abraded surface. It is moistened well with sterile, "normal"

salt solution—6-1,000 of water, and dressed with narrow strips of rubber tissue, and covered with rubber tissue and large quantities of plain gauze saturated with the same solution. The grafts, as few in number and as large as possible for each ulcer, are redressed with fresh gauze saturated with salt solution every forty-eight hours, through small openings in the rubber sheet.

In one week the grafts have established a connected circulation with the live tissues. The very thinnest slice of cuticle possible must be used for the graft. The locality from whence they are taken bleeds but very little, only a slight oozing. Sterilized rubber tissue dressing will heal the surface in forty-eight hours.

The Trendelenburg posture has marked a wonderful progress in pelvic surgery. This posture consists in elevating the hips and trunk to an angle of about 45 degrees, and from fifteen to twenty-four inches higher than the head. This posture causes the pelvic and abdominal viscera to gravitate downward, rendering, upon abdominal incision, the view of the pelvic organs clear, thereby simplifying operations in that cavity.

The trephine has about yielded its place to the chisel, gouge and rougeur forceps in cranial surgery where the bones are to be perforated. The best surgeons in the metropolis prefer and use those instruments instead of the trephine. The operation in expert hands is safer, quicker and the results are superior to the old method.

In obstetrics, the possibilities of accurate diagnosis of the different positions of the fœtus in utero by abdominal palpation is really wonderful. With astonishing exactness the precise presentation can be made by an expert without a vaginal examination. Great advantages will soon flow from this method of diagnosing the presentation when our obstetricians realize the fact that by this method the woman is immune against sepsis being introduced into the vagina and uterus by their fingers.

The strictest asepsis is practiced in obstetrics. The woman receives frequent baths about the time of maturation of gestation. The genitalia are washed and scrubbed with soap and brush. The lying-in apartment and all the bedding and linen are to be scrupulously clean. The accoucheur's hands are thoroughly cleansed and disinfected. A few vaginal examinations only are made, and then when the head is impinging on the perineum. After the child is delivered the placenta is expressed and delivered by the Credé method. After labor is completed the perineum is sutured if lacerated. The blood stains are all entirely removed by thorough antiseptic sponging, and then a large quantity of antiseptic gauze is placed over the vulva and pubes and fastened by a T bandage. The gauze is often removed and replaced by a fresh supply. This kind of antiseptic dressing is kept up until the lochia has ceased.

In post partal hemorrhage if hot intrauterine injections of water have not promptly succeeded, the interior of the uterus is packed with sterile or weakly antiseptic gauze, yard upon yard, until its cavity is completely filled. The vagina is then filled with the guaze, and then a firm T bandage is applied. There can then be no further bleeding; and when the womb regains its tone and the woman her strength, pains are resumed, and after some hours or a day the guaze is expelled, the uterus now contracting well. In this connection it is my pleasure to mention Prof. R. H. M. Dawbarn's new technique in the use of saline infusion to restore flagging vitality in such cases of profound anæmia from post partal hemorrhage or otherwise. It is to my mind the most ingenious and efficient extemporaneous contrivance ever devised by the fertile brain of a surgeon. Briefly stated it is this: A large, ordinary hypodermic needle is inserted into the femoral artery an inch or two below Poupart's ligament as soon as bright blood is seen to well up from within the needle, slip over its base a soft rubber catheter or piece drainage tube which is already attached to a Davidson's syringe, both being filled with warm salt water (heaping teaspoonful to the quart of warm water, 6-1,000). Tie a thread tightly over the catheter, securing it to the base of the needle. Have an abundance of salt water and immerse the syringe entire, with the hand working it, and steadily pump the fluid directly into the arterial current. In explanation it is only necessary to state that the sinking of the syringe in the salt solution is to avoid the possibility of introducing air into the circulation. By this magnificent method Prof. Dawbarn, with the assistance of Dr. H. G. Myers, restored to life on the 17th day of May, 1891, a primipara who was so exsanguinated from post partal hemorrhage as to be unconscious in low muttering delirium, the whole surface deathly cold, and at a glance looked moribund, features pinched, pupils dilated, eyes looked glazed. The case was reported to the New York Academy of Medicine, Surgical Section, Nov. 9th, 1891. He has informed me that he is about to report four additional cases to the *New York Medical Record* which he has treated in the same way.

Progressive physicians, lovers of medical advancement, will ever hail with delight and put into practice all rational innovations that have proven a boon in the prevention and cure of diseases.

